# Flash-Flood Potential Mapping Using Deep Learning, Alternating Decision Trees and Data Provided by Remote Sensing Sensors

**DOI:** 10.3390/s21010280

**Published:** 2021-01-04

**Authors:** Romulus Costache, Alireza Arabameri, Thomas Blaschke, Quoc Bao Pham, Binh Thai Pham, Manish Pandey, Aman Arora, Nguyen Thi Thuy Linh, Iulia Costache

**Affiliations:** 1 Research Institute of the University of Bucharest, 90-92 Sos. Panduri, 5th District, 050663 Bucharest, Romania; romulus.costache@icub.unibuc.ro; 2 National Institute of Hydrology and Water Management, București-Ploiești Road, 97E, 1st District, 013686 Bucharest, Romania; 3 Department of Geomorphology, Tarbiat Modares University, Tehran 36581-17994, Iran; 4 Department of Geoinformatics–Z_GIS, University of Salzburg, 5020 Salzburg, Austria; Thomas.Blaschke@sbg.ac.at; 5 Environmental Quality, Atmospheric Science and Climate Change Research Group, Ton Duc Thang University, Ho Chi Minh City 700000, Vietnam; 6 Faculty of Environment and Labour Safety, Ton Duc Thang University, Ho Chi Minh City 700000, Vietnam; 7 Geotechnical Engineering Deparment, University of Transport Technology, Hanoi 100000, Vietnam; binhpt@utt.edu.vn; 8 University Center for Research & Development (UCRD), Chandigarh University, Punjab 140413, India; manish07sep@gmail.com; 9 Department of Civil Engineering, University Institute of Engineering, Chandigarh University, Punjab 140413, India; 10 Department of Geography, Faculty of Natural Sciences, Jamia Millia Islamia, New Delhi 110025, India; aman.jmi01@gmail.com; 11 Institute of Research and Development, Duy Tan University, Danang 550000, Vietnam; nguyentthuylinh58@duytan.edu.vn; 12 Faculty of Environmental and Chemical Engineering, Duy Tan University, Danang 550000, Vietnam; 13 Faculty of Geography, University of Bucharest, Bd. Nicolae Bălcescu No 1, 1st District, 010041 Bucharest, Romania; iulia.elena.costache@gmail.com

**Keywords:** flash-flood potential index, remote sensing sensors, bivariate statistics, deep learning neural network, alternating decision trees, ensemble models

## Abstract

There is an evident increase in the importance that remote sensing sensors play in the monitoring and evaluation of natural hazards susceptibility and risk. The present study aims to assess the flash-flood potential values, in a small catchment from Romania, using information provided remote sensing sensors and Geographic Informational Systems (GIS) databases which were involved as input data into a number of four ensemble models. In a first phase, with the help of high-resolution satellite images from the Google Earth application, 481 points affected by torrential processes were acquired, another 481 points being randomly positioned in areas without torrential processes. Seventy percent of the dataset was kept as training data, while the other 30% was assigned to validating sample. Further, in order to train the machine learning models, information regarding the 10 flash-flood predictors was extracted in the training sample locations. Finally, the following four ensembles were used to calculate the Flash-Flood Potential Index across the Bâsca Chiojdului river basin: Deep Learning Neural Network–Frequency Ratio (DLNN-FR), Deep Learning Neural Network–Weights of Evidence (DLNN-WOE), Alternating Decision Trees–Frequency Ratio (ADT-FR) and Alternating Decision Trees–Weights of Evidence (ADT-WOE). The model’s performances were assessed using several statistical metrics. Thus, in terms of Sensitivity, the highest value of 0.985 was achieved by the DLNN-FR model, meanwhile the lowest one (0.866) was assigned to ADT-FR ensemble. Moreover, the specificity analysis shows that the highest value (0.991) was attributed to DLNN-WOE algorithm, while the lowest value (0.892) was achieved by ADT-FR. During the training procedure, the models achieved overall accuracies between 0.878 (ADT-FR) and 0.985 (DLNN-WOE). K-index shows again that the most performant model was DLNN-WOE (0.97). The Flash-Flood Potential Index (FFPI) values revealed that the surfaces with high and very high flash-flood susceptibility cover between 46.57% (DLNN-FR) and 59.38% (ADT-FR) of the study zone. The use of the Receiver Operating Characteristic (ROC) curve for results validation highlights the fact that FFPI_DLNN-WOE_ is characterized by the most precise results with an Area Under Curve of 0.96.

## 1. Introduction

In recent decades, climate change and its related phenomena, e.g., flash floods, have had significant negative effects worldwide for both human society and environment [1]. The extreme rainfalls, extreme river discharge values, and therefore the flash-flood risk are characterized by a continuous increasing trend [2]. This trend is also validated by the huge amount of damages that flash floods generate worldwide. Therefore, an increasing number of studies in the literature approaching the subject of flash-flood susceptibility can be also observed [3,4,5,6]. Moreover, the estimation of flood risk and vulnerability became an essential and mandatory procedure which should be included in the Flood Risk Management strategy [7]. In this regard, the Geographic Informational Systems (GIS) and Remote Sensing (RS) techniques represent the necessary tools, which facilitate the spatial modelling and mapping of flash-flood susceptible areas. It is worth emphasizing the crucial role of Remote Sensing sensors in the observation’s campaigns conducted for the identification of areas already affected by flash-flood processes [8]. Thus, without the RS sensors, the correct inventory of the torrential areas, which favor the occurrence of flash flood, will be impossible. Consideration of the previously affected areas and their involvement as input data in more advanced techniques such as machine learning or bivariate statistics, is of a real help to estimate as accurate as possible the flash-flood susceptibility within a specific catchment [9].

In recent years, new techniques and models have been developed by researchers worldwide [10,11,12,13,14,15,16,17,18,19,20]. During the last 6 years, several studies have been individualized regarding the flash-flood susceptibility investigations, which were carried out through the integration of GIS techniques with bivariate statistical models such as: frequency ratio [21], weights of evidence [22], statistical index [23], evidential belief function [24], certainty factor [25], or index of entropy [26]. Another category of methods successfully used in this type of study are those included in Multicriteria Decision Making such as: Technique for Order Preference by Similarity to Ideal Solution (TOPSIS) [27], Analytical Hierarchy Process (AHP) [28], Analytical Network Process (ANP) [29] or Vlse Kriterijuska Optamizacija I Komoromisno Resenje (VIKOR) [30]. Promising results in terms of flash-flood susceptibility were also provided by machine learning models such as: logistic regression [31], naïve bayes [32], artificial neural network [33], random forest [34,35], support vector machine [36], neuro-fuzzy inference system [37], *k*-nearest neighbor [38] or deep learning neural network [39]. The attempts of researchers to combine models from the same category or from different categories to generate ensemble algorithms that are considered much more accurate than the stand-alone ones should also be noted [40]. In this regard, the following examples can be provided: Fuzzy Unordered Rules Induction Algorithm (FURIA) [3], Bayesian-based machine learning models [9], machine learning and multicriteria decision making ensembles [7], machine learning and bivariate statistics ensembles [41].

Taking into account the previously presented aspects, the main purpose of the proposed research work is to estimate the susceptibility to flash floods in the basin of the Bâsca Chiojdului river from Romania. Estimation of flash-flood exposure will be based on the data collected using Remote Sensing sensors and the GIS database and their use in a number of four ensemble models generated by combining bivariate statistics with deep learning neural networks and alternating decision trees. Thus, on the one hand, the Frequency Ratio and Weights of Evidence bivariate statistical models will be used; these being combined with deep learning neural network and alternating decision trees. The construction of Receiver Operating Characteristic (ROC) curve and the calculation of several statistical metrics will ensure the validation of the results and the evaluation of the models’ performances. It is worthwhile to note that the present study is intended to enrich the scientific literature regarding the flash-flood susceptibility assessment by proposing, for the first time in the literature, the combination above mentioned of four machine learning ensemble models with the GIS and remote sensing techniques.

## 2. Study Area

The Bâsca Chiojdului river basin from Romania, on which the present research is focused, has a total area of 340 km^2^. The basin has an elevation which varies from 242 m to 1463 m, and a slope angle with an average value of 12.3°. It should be noted that a percentage of 79% of the total area is characterized by slope angles higher than 7° [42]. The circularity ratio, that is another important feature with a high influence on flash-flood susceptibility, has a value of 0.46, while the river basin concentration time is 7.27 h [21]. The low concentration time highlights a high predisposition of the study area to the flash-flood events. The forest vegetation covers a total percentage of 50%, while in terms of the soil component, the hydrological group B accounts for approximately 41% of the total research area.

The lithology consists mainly of the sedimentary rocks included in the Paleogene and Cretaceous flysch. The climate is characterized by a high continentalism degree, and especially in the warm season, the heavy rainfalls often lead to severe flash-flood phenomena. Due to the geographical characteristics of the Bâsca Chiojdului river basin, the socio-economic elements located across its territory suffered material losses following the flash-flood propagation. The most important flash-flood event occurred in 1975, when the maximum discharge value (300 m^3^/s) of the Bâsca Chiojdului river reached the historical maximum [42]. More information regarding the main flash floods that occurred across the study area, as well as the damages caused by these phenomena, can be found in the research works carried out by: Prăvălie and Costache [42], Costache et al. [23], Zarea and Gheorghe [43], Prăvălie and Costache [44].

## 3. Data

In order to carry out the present study, data consisting of torrential areas polygons and flash-flood predictors were gathered.

### 3.1. Torrential Area Inventory and Sampling

**Figure 1:**
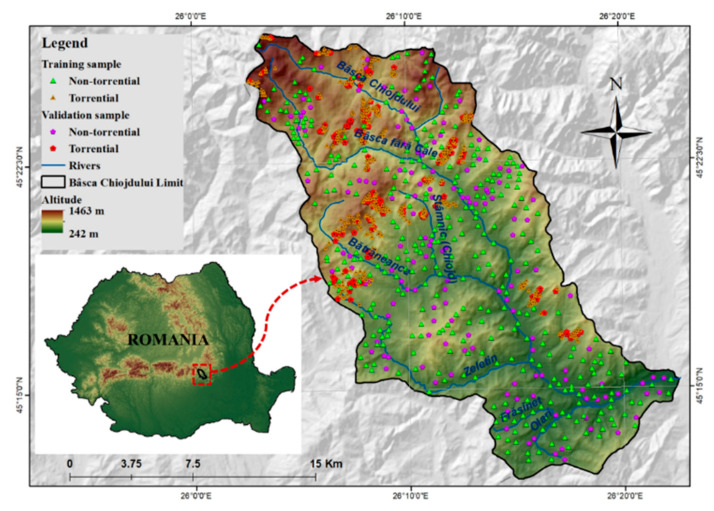
Study area location.

The inventory of surfaces previously affected by a specific process is essential for an accurate prediction of the areas where that phenomenon can occur in the future [45]. In the present research work, we consider the torrential surfaces as the spatial indicator for the areas with a high susceptibility for flash-flood genesis. In order to identify, as accurate as possible, the areas affected by torrential phenomena, analysis of the images provided by the Remote Sensing sensors was mandatory. This fact highlights the crucial role that this type of sensor has in the analysis of natural hazard susceptibility. Thus, using the Google Earth imagery a total area of 34 km^2^ was delimited. These surfaces were created by the accelerated surface runoff occurring on the slopes. The manner in which these surfaces are delineated is described in the study carried out by Costache [46]. According to Costache and Zaharia [8], the torrential areas are defined as the areas characterized by the unified presence of torrential microform of relief such as ravines and gullies, which are generated by surface runoff. They are located in the upper part of the river basin, where the absence of vegetation and the high slopes favor the production of such phenomena. In order to be taken into account in the present study, a sample of 481 points representing locations where the torrential runoff took place was extracted from the entire delimited area. Moreover, another sample of 481 points was placed within the study area, representing points without torrential processes (Figure 1). Both torrential pixels and non-torrential pixels were divided into training (70%) and validating (30%) samples. This division was necessary in order to train the models and then to validate the results regarding the susceptibility to flash floods.

### 3.2. Flash-Flood Predictors

**Figure 2:**
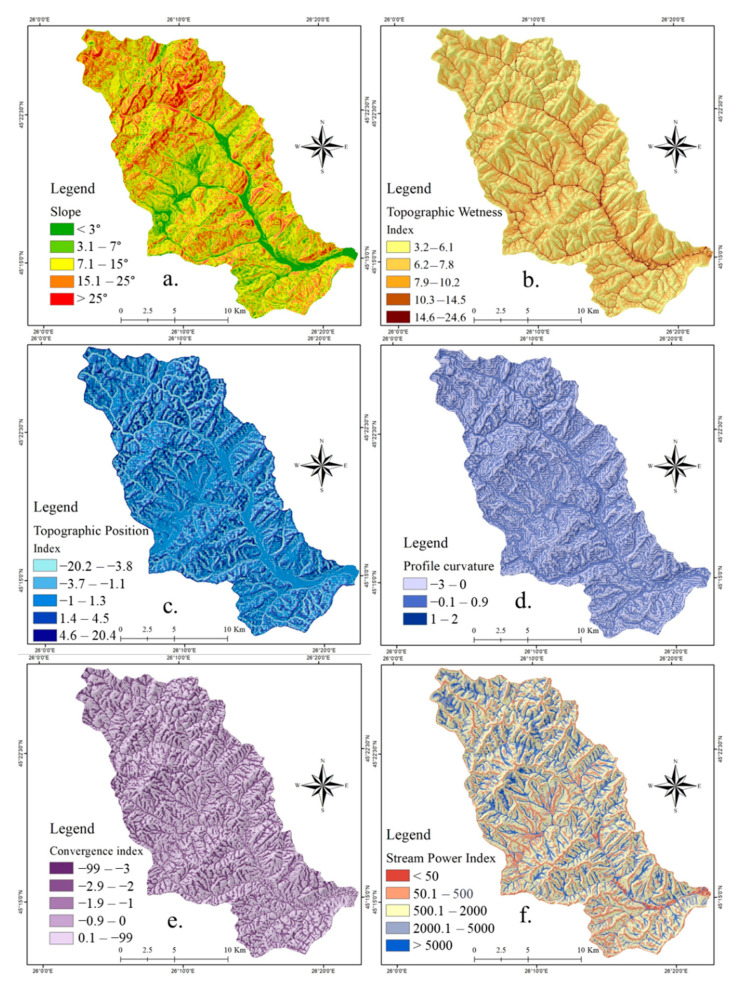
Flash-flood predictors: (**a**) Slope; (**b**) Topographic Wetness Index (TWI); (**c**) Topographic Position Index (TPI); (**d**) Profile curvature; (**e**) Convergence index; (**f**) Stream Power Index (SPI).

**Figure 3:**
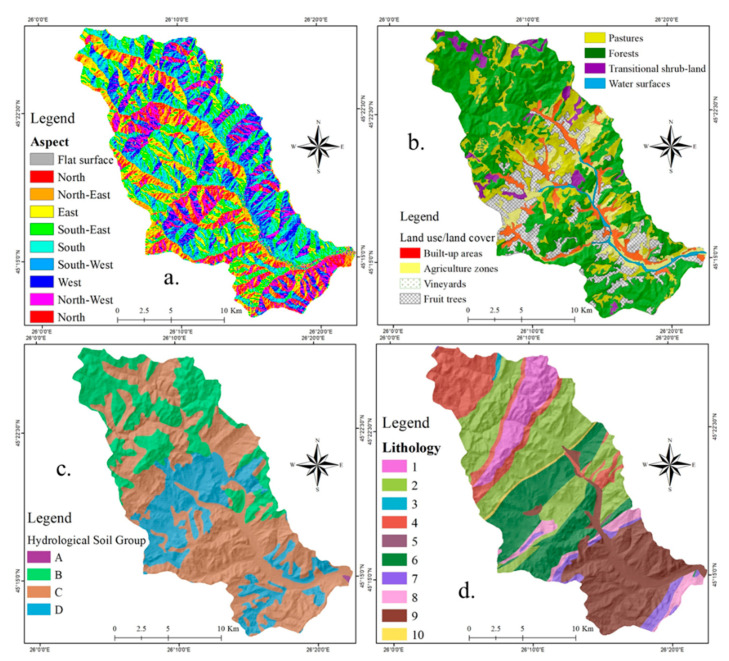
Flash-flood predictors: (**a**) Aspect; (**b**) Land use; (**c**) Hydrological soil groups; (**d**) Lithology.

For the realization of this study, a number of 10 flash-flood conditioning factors were taken into account. Their main properties are described in the following lines. **Slope angle** was calculated using the Digital Elevation Model (DEM) taken from Shuttle Radar Topographic Mission (SRTM) 30 m database and processed in ArcGIS 10 software. A high value of slope angle will influence in a positive water runoff velocity, while the low values of the same parameter will be restrictive for the surface runoff occurrence [41]. For the study area, the map of slope angle was designed by splitting its range of values into five classes as follows: <3°; 3°–7°; 7.1°–15°; 15.1°–25°; >25° (Figure 2a). Another water surface runoff predictor is represented by the **Topographic Wetness Index (TWI)** calculated by the DEM processing in SAGA GIS 2.1.0. The algorithm used to calculate this index requires the use of the area upslope to each pixel and the tangent value of the slope value recorded in the same pixel [38]. The generation of TWI map was possible following the partition of its values into the next five classes using *Natural Breaks* method: 3.15–6.1, 6.11–7.78, 7.79–10.21, 10.22–14.5, 14.51–24.59 (Figure 2b). **Topographic Position Index (TPI)** is a mandatory flash-flood predictor which should be involved in the susceptibility related studies because its values emphasize the altitude difference between the location of a specific point and its neighboring area [47]. This important morphometric indicator was achieved at a spatial resolution of 30 m and its values ranging from −20 to 20 were divided into the next five classes using *Natural Breaks* method: (−20)–(−3.8), (−3.7)–(−1.1), (−1.1)–1.3, 1.4–4.5, 4.6–20 (Figure 2c). **Profile curvature** is mainly used to delineate the surfaces on which an accelerated surface runoff is manifested from those on which a decelerated surface runoff occurs [48]. According to the literature [23], positive profile curvature is characteristic for areas with a decelerated water runoff, while the negative values show the surfaces that increase the water runoff velocity. Across the study area, the profile curvature was classified into the following three intervals: (−3)–0, 0.1–0.9, 1–2 (Figure 2d). The ability of **convergence index** morphometric factor consists of the differentiation of the areas belonging the river valleys from those which are situated along the interfluvial lines. This index, achieved by DEM processing in SAGA GIS 2.1.0, was classified according to the literature: (−99)–(−3), (−2.9)–(−2), (−1.9)–(−1), (−0.9)–0, 0–99 (Figure 2e). **Stream Power Index (SPI)** is another morphometric factor that is generated in SAGA GIS 2.1.0 based on the values of upslope region that drains into a pixel and the tangent applied to the slope angle [49]. This predictor, which shows the capacity of the river for sediment transport, was mapped using the following classes values: <50, 50–500, 501–2000, 2001–5000, >5000 (Figure 2f). **Slope aspect** (Figure 3a) is the seventh morphometric index taken into account for the present research. The slope orientation has a big influence in the surface runoff process because the humidity condition will vary due to the different quantity of solar radiation [50]. The slope aspect predictor was derived from the DEM.

Land use, which is the main interface between the torrential rainfalls and the ground surface, has an important influence on the runoff velocity [51]. For the present study, the land use layer was taken from the **Corine Land Cover** 2018 database. According to Figure 3b, a number of eight land use categories were delineated within the study area perimeter. **Hydrological soil group** was considered as a flash-flood predictor in the present research due to its incontestable influence on vertical infiltration of water in the ground [52]. Within the Bâsca Chiojdului cathcment, all of the four hydrological soil groups are present (Figure 3c). A similar contribution, as soil groups, in flash-flood genesis is held by the **lithological groups**. In the area of the Bâsca Chiojdului catchment, a total of 10 lithological groups can be found (Figure 3d).

## 4. Methods

**Figure 4:**
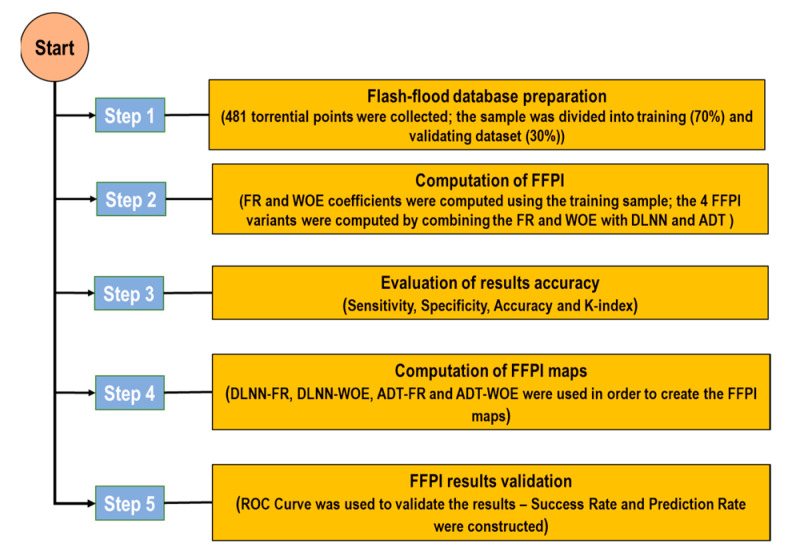
Flowchart of the methodological steps applied in this research.

The main steps of the methodological workflow are synthetically described in Figure 4.

### 4.1. Linear Support Vector Machine (LSVM) for Feature Selection

In a study that aims to estimate the qualitative flash-flood susceptibility, it is imperative to analyze the predictive ability of flash-flood conditioning factors in order to see if they all manage to contribute to some extent to the genesis of flash floods. In the present research paper, the evaluation of the prediction ability of flash-flood predictors was determined using Linear Support Vector Machine (LSVM). This method is widely used because it is able to remove redundant and irrelevant information from input data [53]. The following equation is used to compute the predictive ability through LSVM algorithm [54]:

(1) $$f\left(x\right)=sign\left(C^{T}*i+j\right)$$

where *C^T^* is equal to the inverse of weight matrix attributed to each flash-flood predictor, *i* = (*i*1, *i*2,*…*, *i*11) is the vector containing the ten flash-flood predictors, *j* is equal to the offset value calculated from the hyper-plane origin [5].

This algorithm was applied with the help of Weka 9.3 software.

### 4.2. Weights of Evidence (WOE)

The bivariate statistics model represented by Weights of Evidence (WOE) is a very frequently used algorithm involved in the studies focused on natural hazards predisposition evaluation [25]. In this study, the WOE model is used to calculate the weight that each factor class/category has in relation to the genesis of the flash-flood process. In order to derive the WOE coefficients, first, computing the positive (*W*^+^) and negative (*W*^−^) weights is required. The positive weight highlights the association between a factor class/category and the torrential points, while the negative weight indicates the absence of this spatial association [21]. The following relations should be employed in the weights computation [55]:

(2) $$W^{+}=\ln\frac{P\{B|S\}}{P\{B|\bar{S}\}}$$

(3) $$W^{-}=\ln\frac{P\{\bar{B}|S\}}{P\{\bar{B}|\bar{S}\}}$$

where: *W*^+^—positive weight, *W*^−^—negative weight, *P*—the probability, *B*—the presence of flash-flood predictor, $\bar{B}$ —the absence of flash-flood predictor, S—the presence of torrential pixels, $\bar{S}$ —the absence of torrential pixels.

The final WOE coefficients can be derived using the next equation [56]:

*Wf* = *Wplus* + *W*min*total* – *W*min (4)

where: *Wplus*—positive weight of a class factor, *W*min—negative weight of a class factor, *W*min*total*—the total of all negative weights in a multiclass map.

The final WOE values will be used as input data into the Deep Learning and Alternating Decision Tree models through which the flash-flood susceptibility will be determined.

### 4.3. Frequency Ratio (FR)

Frequency Ratio (FR) is the second bivariate statistical model which will be employed in order to prepare the input data in the Deep Learning and Alternating Decision Tree algorithms. The FR model consists of the calculation of the ratio between the sum of torrential pixels within a specific category of predictor, and the sum of torrential pixels within the entire study zone. The following relation can be used to estimate the FR coefficients [57]:

(5) $$FR=\frac{\frac{Np\left(LXi\right)}{\sum_{i=1}^{m}Np\left(LXi\right)}}{\frac{Np\left(Xj\right)}{\sum_{j=1}^{n}Np\left(Xj\right)}}$$

where: *FR*—the frequency ratio of class *i* of factor *j*; *Np*(*LXi*)—the number of pixels with torrentiality within class *i* of factor variable *X*; *Np*(*Xj*)—the number of pixels within factor variable *Xj;* m—the number of classes in the factor variable *Xi*; *n*—the number of factors in the study area.

### 4.4. Deep Learning Neural Network (DLNN)

Besides one hidden layer neural networks, the Deep Learning Neural Network (DLNN) is characterized by a feed-forward architecture which contains more than one hidden layer [58]. Due to this fact, DLNN model is considered better than the simple neural network in terms of complex classification problems [59]. In the DLNN structure, the information from the input layer will be transmitted to the hidden layers where it is processed and then forwarded to the output layer. Further, the backpropagation algorithm will be employed to send back the error from the output layer to the input layer [60]. The training procedure of DLNN, which is a type of fee-forward neural network, is ensured by the application of Rectified Linear Unit (ReLU) activation function [61]. This function, which is able to reduce the vanishing gradient, is expressed as follows:

(6) $$r\left(x\right)=\{\begin{array}{c} |x\;if\;x>0\\ |0\;if\;x\leq 0 \end{array}=\max(0,x)$$

where *x* is the input signal transmitted to neuron, while *r* is the ReLU function.

The derivate associated to the ReLU function, which are required by the back-propagation algorithm, can be calculated using the following relation:

(7) $$r’\left(x\right)=\{\begin{array}{c} |1,\;x>0\\ |0,\;x\leq 0 \end{array}$$

It should be remarked that the cross-entropy function is also involved in the training procedure because it helps the DLNN to achieve a higher degree of accuracy [62]. The cross-entropy is mathematically described using the next equation:

(8) $$E=-\frac{1}{N}\overset{N}{\underset{n=1}{\sum }}M\ln\left(P\right)+\left(1-M\right)\ln\left(1-P\right)\;$$

where *N* is the total number of records in training sample; *M* is the predictor values, while *P* is the predicted values.

The adaptive momentum (Adam) prediction model, implied in the stochastic optimization process, is used to complete the training process of DLNN. Through the Adam model, the first and second moments could be computed via the exponential moving averages highlighted through the next relations [63]:

(9) $$m_{t}=\beta_{1}m_{t-1}+\left(1-\beta_{1}\right)g_{t}$$

(10) $$v_{t}=\beta_{2}v_{t-1}+\left(1-\beta_{2}\right)g_{t}^{2}$$

where *m* and *v* are the values of the moving averages, *g* represents current mini-batch gradient, β is new hyper-parameters computed via the algorithm.

In order to apply the DLNN-FR and DLNN-WOE ensembles, the specific lines of code were written in R programming language. More specifically, the Keras and Lime package from R Studio were used in this regard. 

### 4.5. Alternating Decision Tree

Alternating Decision Tree (ADT) model is an ensemble of the decision tree and boosting method [64]. ADT structure has a lower complexity than decision tree models such as Rotation Forest, Classification and Regression Tree or Random Forest [65]. ADT model uses a natural extension of decision tree and voted stumps and is formed by prediction alternate layers and nodes of decision [66]. Within the ADT algorithm, the decision nodes will specify the predicate condition; meanwhile the prediction nodes will be characterized by a single number [65].

Let *c*_1_ be the value of a precondition, *c*_2_ the value of a base condition, and *a* and *b* the values of two real numbers; then *a* and *b* will be computed using the relations [67]:

(11) $$a=0.5*\ln\frac{W_{+}(c_{1}\cap c_{2})}{W_{-}(c_{1}\cap c_{2})},\;b=0.5*\ln\frac{W_{+}(c_{1}\cap \bar{c_{2}})}{W_{-}(c_{1}\cap \bar{c_{2}})}$$

where *W* denotes the sum of the values from any prediction node, and the best *c*_1_ and *c*_2_ are estimated by minimizing the *Z_t_* (*c*_1_, *c*_2_), determined as follows:

(12) $$z_{t}\left(c_{1}c_{2}\right)=2\sqrt{W_{+}(c_{1}\cap c_{2})*W_{-}(c_{1}\cap c_{2})}+\sqrt{W_{+}(c_{1}\cap \bar{c_{2}})*W_{-}(c_{1}\cap \bar{c_{2}})}+W\left(\bar{c_{2}}\right)$$

The ADT-FR and ADT-WOE ensembles were run and implemented in Weka software.

### 4.6. Model Performance and Results Validation

#### 4.6.1. Statistical Measures

At the first stage, the models’ performance assessment will consist of the computation of the next statistical metrics: specificity, sensitivity, accuracy, kappa index. The aforementioned indices will be computed using the next mathematical relations:

(13) $$k=\frac{p_{o}-p_{e}}{1-p_{e}}$$

(14) $$Sensitivity=\frac{TP}{TP+FN}$$

(15) $$Specificity=\frac{TN}{FP+TN}$$

(16) $$Accuracy=\frac{TP+TN}{TP+FP+TN+FN}$$

where *TP* (True Positive) and *TN* (True Negative) are the sum of points that will be correctly classified, *FP* (False Positive) and *FN* (False Negative) are the sum of points erroneously classified; *k* is kappa coefficient, *p_o_* is the sum of initially established torrential pixels, and *p_e_* is the sum of predicted torrential pixels.

#### 4.6.2. ROC Curve

The second stage of results validation implied the application of the ROC curve and Area Under Curve (AUC) to measure the model performance. An AUC closer to 1 will highlight a performant model, while the values near to 0 will indicate a weak prediction ability of the models [68,69]. The Success Rate will represent a first form of ROC curve which will be constructed with the training samples, while the Prediction Rate is the second variant of ROC curve which will be designed with the help of validation sample. The AUC values will be determined using the next formula:

(17) $$AUC=\frac{\left(\sum TP+\;\sum TN\right)}{\left(P+N\right)}$$

where *P* is the sum of points having torrential phenomena and *N* is the sum of non-torrential points.

## 5. Results

### 5.1. Feature Selection Using LSVM

**Figure 5:**
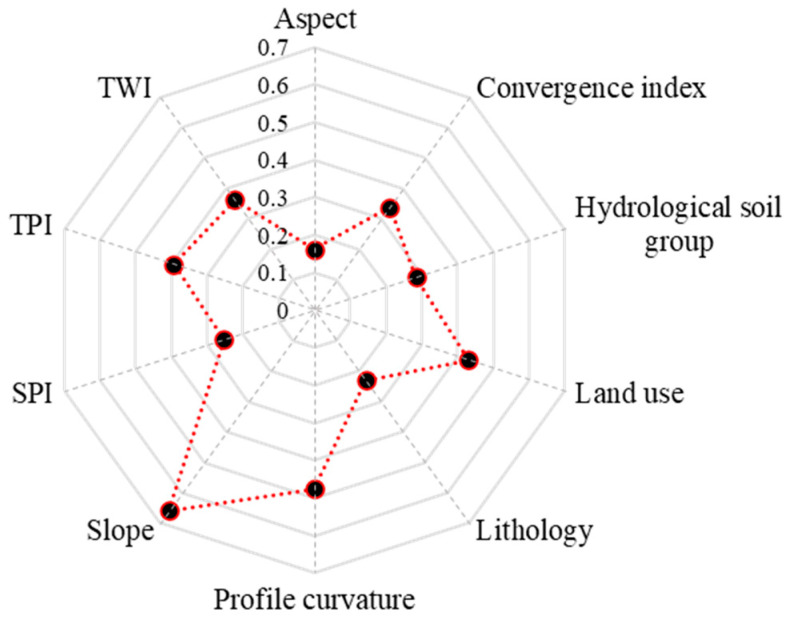
Linear Support Vector Machine (LSVM) scores assigned to flash-flood predictors.

According to the results achieved through Weka software, the application of LSVM provided the next scores: slope (0.659), profile curvature (0.476), land use (0.429), tpi (0.394), twi (0.362), convergence index (0.338), hydrological soil group (0.283), spi (0.253), lithology (0.231) and aspect (0.162) (Figure 5).

### 5.2. FR and WOE Coefficients

**Figure 6:**
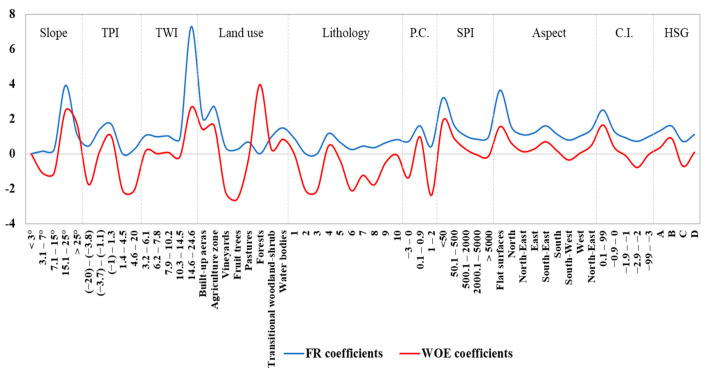
Distribution of FR and WOE coefficients within the classes of flash-flood predictors.

The values of FR and WOE coefficients are inserted in Table 1. The largest value of FR coefficients (7.295) was achieved by TWI class between 14.6 and 24.6, followed by slope class between 15 and 25° (3.925), SPI values lower than 50 (3.205), built-up areas land use category (2.715) and TPI class between −1 and 1.3 (1.695) (Figure 6). In terms of WOE weights, the highest score was assigned to built-up areas land use category (3.96), followed by TWI class between 14.6 and 24.6 (2.67), slope class between 15 and 25° (2.48), SPI values lower than 50 (1.88) and TPI class between −1 and 1.3 (1.39).

In order to be used as input in ADT and DLNN models, the FR and WOE values were normalized between 0 and 1.

### 5.3. Models Performance Assessment

The configuration, in terms of the hardware and software environments, that was required for the computational modelling, is presented in Table 2.

It is mandatory that before the final mapping of flash-flood potential, the model’s performance must be evaluated in order to verify its reliability in the methodological process. Thus, in terms of the training dataset, the DLNN-WOE ensemble achieved the highest accuracy (0.985), followed by DLNN-FR (0.982), ADT-FR (0.923) and ADT-WOE (0.92). In terms of the validating sample, the highest accuracy was achieved by DLNN-WOE (0.92), followed by DLNN-FR (0.903), ADT-WOE (0.896) and ADT-FR (0.878) (Table 3).

### 5.4. Results of Machine Learning Ensembles

#### 5.4.1. DLNN-FR and DLNN-WOE Results

**Figure 7:**
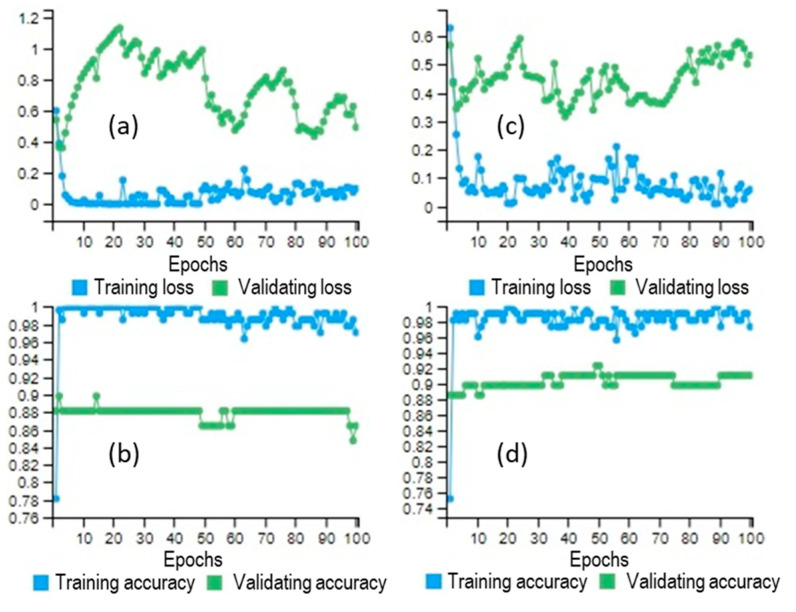
DLNN based ensemble running outputs (**a**) Training and Validating loss of DLNN-FR; (**b**) Training and Validating accuracy of DLNN-FR; (**c**) Training and Validating loss of DLNN-WOE; (**d**) Training and Validating accuracy of DLNN-WOE).

The DLNN based ensembles were trained by establishing the maximum number of epochs to 100 (Figure 7).

**Figure 8:**
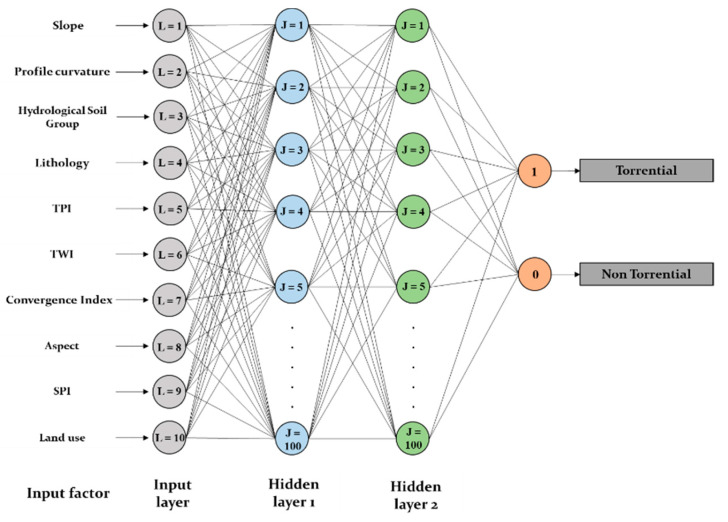
Deep Learning Neural Network architecture.

Figure 7 highlights the variability of loss and model accuracy according to the epochs number and also for both training and validating samples. Particularly, in the case of the DLNN-FR model, the best performances were achieved with the following model parameters: a number of two hidden layers; a maximum number of 100 hidden neurons in each hidden layer; a dropout rate of 0.3; a batch size value of 5 and a validation split of 0.3. The same number of hidden layers and neurons was used also in the case of DLNN-WOE, while the other parameters have the following value: a dropout rate of 0.4; a batch size of 4 and a validation rate of 0.2. The architecture of the DLNN-based ensembles are represented in Figure 8.

**Figure 9:**
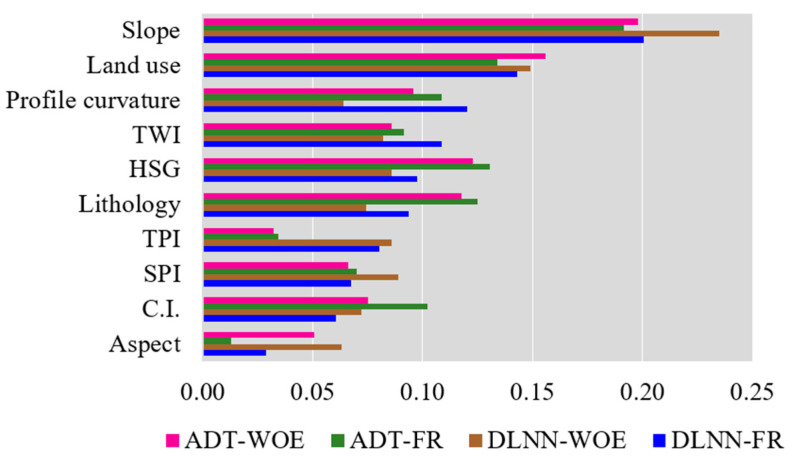
Flash-flood predictors importance.

The next step in the flash-flood susceptibility computation process is the derivation of the flash-flood predictor’s importance. In terms of DLNN-FR, the highest importance was assigned to slope factor (0.2). On the second-place rank, land use (0.143), followed by profile curvature (0.12), TWI (0.109), hydrological soil group (0.097), lithology (0.094), TPI (0.08), SPI (0.067), convergence index (0.061) and aspect (0.029) (Figure 9). The application of DLNN-WOE revealed that the most important factor was slope (0.235), and is followed by land use (0.149), SPI (0.089), hydrological soil group (0.086), TPI (0.086), TWI (0.082), lithology (0.074), convergence index (0.072), profile curvature (0.064) and aspect (0.063).

**Figure 10:**
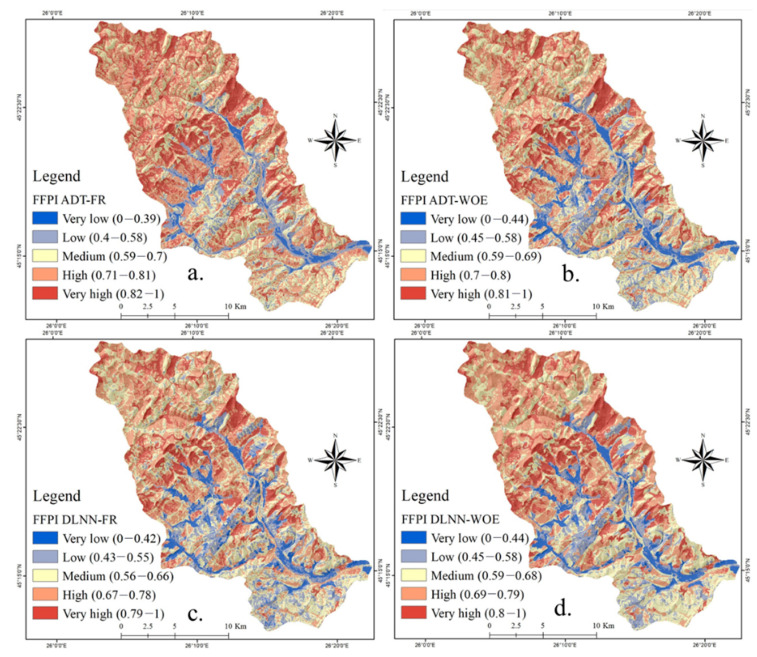
Flash Flood Potential Index (**a**) DLNN-FR; (**b**) DLNN-WOE; (**c**) ADT-FR; (**d**) ADT-WOE.

**Figure 11:**
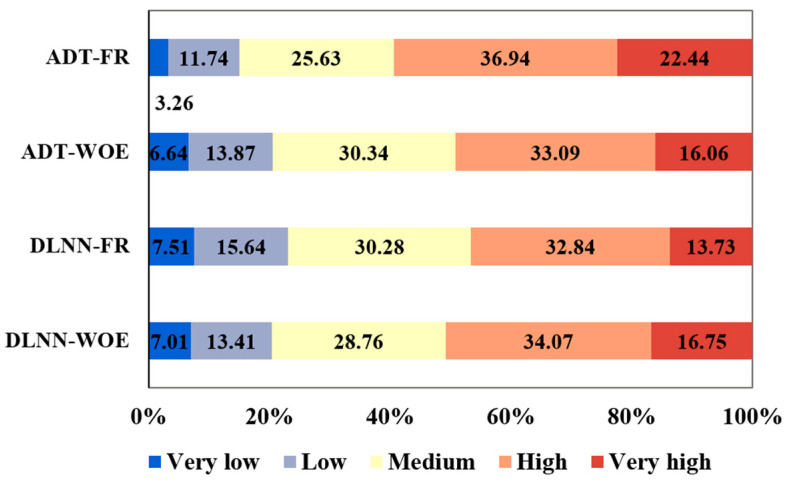
Flash-Flood Potential Index (FFPI) classes weights.

The weights of flash-flood predictors were used in ArcGIS map algebra in order to derive the flash-flood potential index values. All the Flash-Flood Potential Index (FFPI) results, with values between 0 and 1, were reclassified in five classes using Natural Breaks method. In terms FFPI_DLNN-FR_, the very low flash-flood potential values cover around 7.5% of the study area and range between 0 and 0.42 (Figure 10a). The low flash-flood potential appears on around 15.6% of Bâsca Chiojdului river catchment and has values ranging from 0.43 and 0.55. It should be remarked that these values are mainly spread on the southern half of the area. The medium flash-flood potential has a span of 30.28% of the entire territory (Figure 11) and is characterized by FFPI_DLNN-FR_ between 0.56 and 0.66. These values are uniformly distributed across the study zone. The high and very high flash-flood potential appears on areas with FFPI_DLNN-FR_ higher than 0.67 and covers approximately 46.57% of the research area. This potential degree is mainly present in the northern half of Bâsca Chiojdului river basin.

In terms of FFPI_DLNN-WOE_, the very low flash-flood potential is characteristic for a percentage of 7% from the entire study perimeter, while the low values of the same indicator cover an area of 13.41% of the total territory. Ranging from 0.59 to 0.68 (Figure 10b), the medium flash-flood potential spans accross approximately 28.76% of the Bâsca Chiojdului river catchment. High and very high flash-flood susceptibility has values of FFPI_DLNN-WOE_ higher than 0.69 and is spread over more than 50% of the research zone. It should be noted that the areas delineated through DLNN-WOE have a lower degree of fragmentation than the areas delineated by DLNN-FR.

#### 5.4.2. ADT-FR and ADT-WOE Results

**Figure 12:**
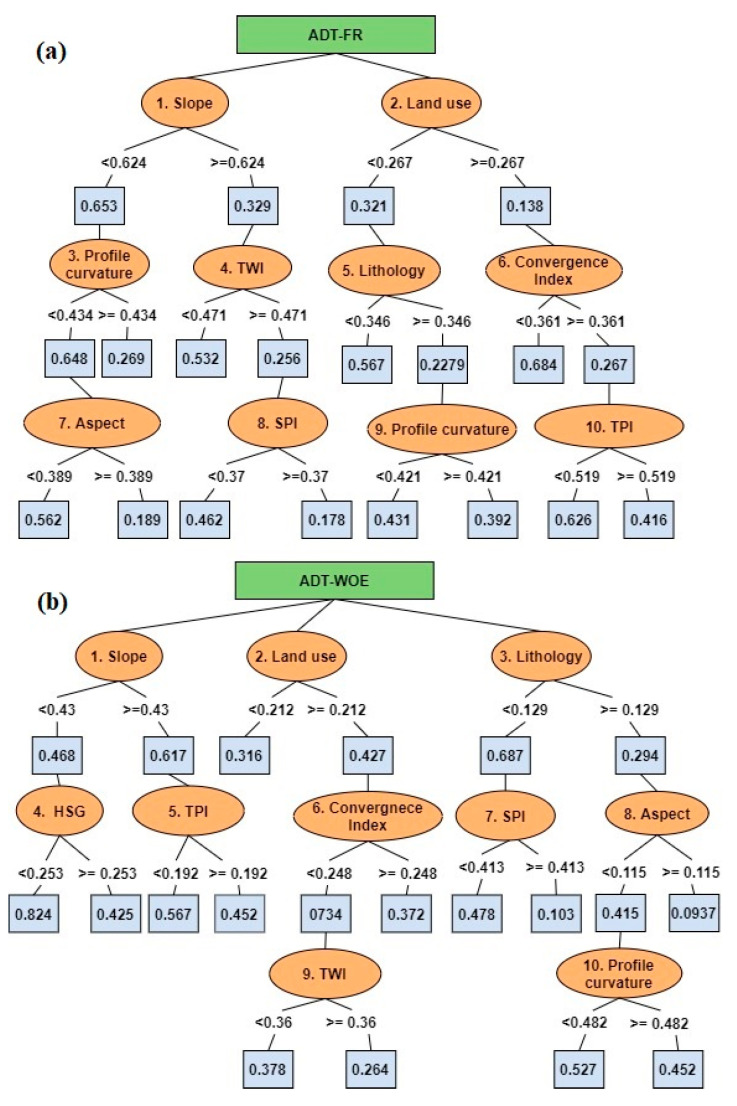
Optimally pruned Decision Tree Structure for ADT based ensembles ((**a**) ADT-FR and (**b**) ADT-WOE ensembles).

A trial procedure was applied in order to determine the best parameter associated with the highest accuracy of ADT-FR and ADT-WOE for both training and validating samples. Thus, in terms of ADT-FR, the highest accuracies (0.923 for training and 0.878 for validating) were achieved after 23 iterations, while in terms of ADT-WOE the best accuracies (0.92 for training and 0.896 for validating) were determined after a number of 28 iterations (Table 4). Once the best parameters were determined, the optimally pruned decision trees were constructed (Figure 12a,b) and the flash-flood predictors importance were calculated.

Therefore, in terms of ADT-FR, the highest importance was assigned to slope factor (0.191). On the second-place rank land use (0.134), followed by hydrological soil group (0.131), lithology (0.125), profile curvature (0.108), convergence index (0.102), TWI (0.091), SPI (0.07), TPI (0.034) and aspect (0.013) (Figure 9). The application of ADT-WOE revealed that the most important factor was slope (0.198), and is followed by land use (0.156), hydrological soil group (0.123), lithology (0.117), profile curvature (0.096), TWI (0.086), convergence index (0.075), SPI (0.066), aspect (0.051) and TWI (0.032).

As in the case of the previous two ensembles, the FFPI_ADT-FR_ and FFPI_ADT-WOE_ were calculated. In terms FFPI_ADT-FR_, the very low flash-flood potential spans around 3.26% of the study area and has values between 0 and 0.39 (Figure 10c). The low flash-flood potential is distributed on around 11.74% of the Bâsca Chiojdului river catchment and has values ranging from 0.4 to 0.58. The medium flash-flood potential spans 25.63% of the entire territory and has values between 0.59 and 0.7 (Figure 10c). The high and very high flash-flood potentials appear on areas with FFPI_ADT-FR_ higher than 0.71 and cover approximately 59.38% of the research area. In terms of FFPI_ADT-WOE_, the very high flash-flood potential covers 6.64% of the entire study perimeter, while the low values are spread over 13.87% of the total territory. With values from 0.59 to 0.69 (Figure 10d), the medium flash-flood potential occurs over 30.34% of the Bâsca Chiojdului river catchment. The high and very high flash-flood potential indices have values higher than 0.7 and account for almost 50% of the study zone.

### 5.5. Results Validation Using ROC Curve

**Figure 13:**
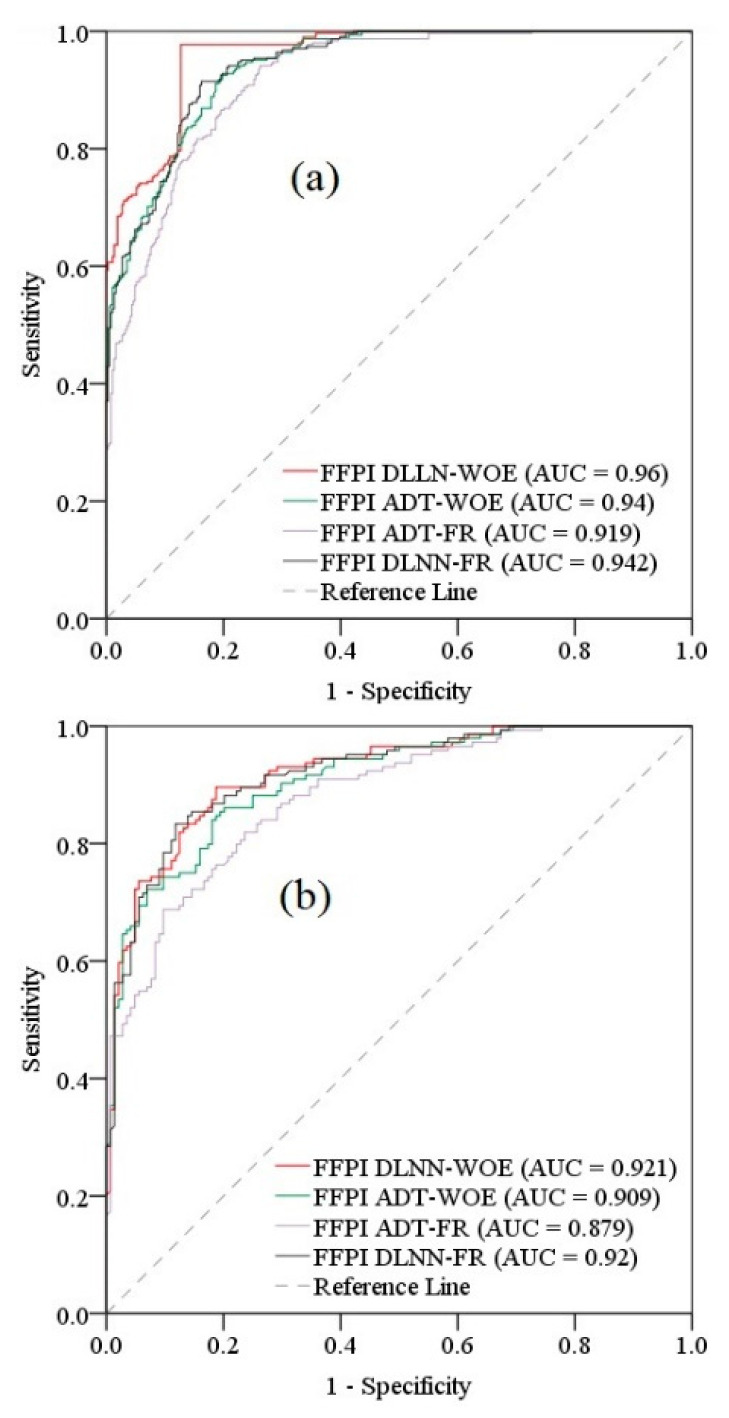
Receiver Operating Characteristic (ROC) curve (**a**) Success Rate; (**b**) Prediction Rate.

The validation of the FFPI results provided by each ensemble model was carried out using the ROC curve method. Thus, in the case of the Success Rate, the highest performance was achieved by FFPI_DLNN-WOE_ with an AUC of 0.96, being followed by FFPI_DLNN-FR_ (AUC = 0.942), FFPI_ADT-WOE_ (AUC = 0.94) and FFPI_ADT-FR_ (AUC = 0.919) (Figure 13a). If we analyze the Prediction Rate outcomes, it can be seen that the same FFPI_DLNN-WOE_ indicator achieved the highest performance (AUC = 0.921), followed by FFPI_DLNN-FR_ (0.92), FFPI_ADT-WOE_ (0.909) and FFPI_ADT-FR_ (AUC = 0.879).

## 6. Discussions

With the undeniable advancement of technology, there are more and more possibilities to monitor the dangerous phenomena that occur on the Earth’s surface. In this regard, it is worth remembering the rapid advance of observation techniques of the terrestrial surface by means of remote sensing sensors—with the help of which, the surfaces affected by natural hazards can be observed.

Thus, the present paper used images taken with the help of these sensors to identify the areas already affected by the torrential runoff from the Earth’s surface. It should be mentioned that the most accurate identification of these areas is essential in obtaining results with high accuracy and which can be further used by the competent authorities in risk assessment and in adopting the most appropriate measures to reduce future damage caused by these hazards. Thus, by analyzing the images provided by remote sensing sensors, on the river basin of the river Bâsca Chiojdului, areas affected by torrential runoff totaling a total area of 34 km^2^, representing about 10% of the entire study area, were identified. Furthermore, in order to capitalize on the delimited surfaces, a sample of about 481 was generated, taking a sample of points affected by torrential phenomena transposed into relief microforms such as ravines. In order to ensure the correctness of the modelling results, another sample of 481 points was generated from the areas where the torrential phenomena did not take place; the entire data set being then divided into training and validating data. The values of 10 flash-flood predictors were also used as input data. It should be noted that Remote Sensing sensors also played a crucial role in generating 8 of the 10 flash-flood predictors. Thus, all morphometric parameters were derived from the digital terrain model taken from the SRTM database, 30 m which was acquired using radar techniques. In addition, the land use, taken from the Corine Land Cover 2018 database, was generated by the supervised classification of the images provided by the Remote Sensing sensors.

Data on the presence of phenomena and the values of the main predictors of flash-flood genesis were included in two of the state-of-the-art machine learning models represented by Deep Learning Neural Networks and Alternating Decision Trees. These two models are recommended due to the very good results they provided following their application in previous studies on the estimation of susceptibility to natural hazards [64,65]. For a higher degree of results objectivity, it was decided to process the training sample by assigning some coefficients using the bivariate methods statistics, Frequency Ratio and Weights of Evidence. This method has proven to be very useful in previous studies [31,41] where the initial data were processed with bivariate statistics algorithms.

The combination of DLNN with WOE proved to be the most efficient because the accuracy achieved during the training process exceeded 98%, while ROC curve applied to the final product FFPI_DLNN-WOE_ showed a maximum AUC of 0.96. This value of AUC exceeds the value obtained by Costache et al. [23], when, by applying the hybrid combination between Multilayer Perceptron (MLP) and Statistical Index, for the same study area and for the FFPI calculation, a maximum AUC value of 0.94 was obtained. These results confirm the findings from the literature according to which DLNN, whose architecture includes several hidden layers, is able to surpass the MLP performances whose architecture includes a single hidden layer [42]. Moreover, the MLP performance from the previous study was exceeded by the DLNN-FR ensemble model, characterized by an AUC of 0.942. Overall, in the Bâsca Chiojdului basin, the models showed a percentage of the high and very high flash-flood potential between 46.57% (DLNN-FR) and 59.38% (ADT-FR).

## 7. Conclusions

In light of the continuous increase in the flash-flood events’ frequency, the present research work proposed a workflow through which the areas susceptible to flash floods are identified based on remote sensing and GIS data included in Deep Learning and Alternating Decision Trees ensembles. Thus, using 418 torrential and 481 non-torrential locations along with 10 flash-flood predictors, the Flash-Flood Potential Index was determined across the Bâsca Chiojdului river basin. Using as input data the FR and WOE coefficients, the FFPI was computed using the following four ensembles: DLNN-FR, DLNN-WOE, ADT-FR and ADT-WOE. As was expected, the slope angle and land use resulted in being the most important flash-flood predictors. The highest results accuracy was achieved by the DLNN-WOE model which is characterized by an AUC–ROC curve of 0.985. The percentage (59.38%) of high and very high FFPI classes was revealed by the application of ADT-FR ensemble.

The main novelty of this study is represented by the application for the first time in the literature of the four ensemble models for determining flash-flood potential index values.

This work is of real importance for the governmental authorities which can use the results in order to improve the measures taken to mitigate the negative effects of flash-flood hazards within the study area.

## Figures and Tables

**Table 1:** FR and WOE coefficients.

Factor	Class	FR	FR Standardized Coefficients	WoE Coefficients	WoE Standardized Coefficients
**Slope**	<3°	0.000	0.000	0.000	0.307
	3.1–7°	0.152	0.039	−1.100	0.000
	7.1–15°	0.245	0.062	−1.100	0.000
	15.1–25°	3.925	1.000	2.480	1.000
	>25°	1.125	0.287	1.720	0.788
**TPI**	(−20)–(−3.8)	0.435	0.257	−1.740	0.116
	(−3.7)–(−1.1)	1.415	0.835	0.160	0.727
	(−1)–1.3	1.695	1.000	1.010	1.000
	1.4–4.5	0.000	0.000	−2.100	0.000
	4.6–20	0.245	0.145	−2.100	0.000
**TWI**	3.2–6.1	1.055	0.030	0.160	0.116
	6.2–7.8	0.975	0.017	0.010	0.063
	7.9–10.2	1.025	0.025	0.080	0.088
	10.3–14.5	0.865	0.000	−0.170	0.000
	14.6–24.6	7.295	1.000	2.670	1.000
**Land use**	Built-up areas	2.035	0.750	3.960	1.000
	Agriculture zone	2.715	1.000	1.610	0.642
	Vineyards	0.365	0.134	−2.190	0.063
	Fruit trees	0.245	0.090	1.390	0.608
	Pastures	0.675	0.249	−0.300	0.351
	Forests	0.000	0.000	−2.600	0.000
	Transitional woodland-shrub	0.965	0.355	0.270	0.438
	Water bodies	1.485	0.547	0.840	0.524
**Lithology**	1	0.895	0.768	0.000	0.745
	2	0.000	0.000	−2.100	0.000
	3	0.000	0.000	−2.100	0.000
	4	1.165	1.000	0.450	0.904
	5	0.665	0.571	−0.350	0.621
	6	0.245	0.210	−2.100	0.000
	7	0.435	0.373	−1.230	0.309
	8	0.355	0.305	−1.770	0.117
	9	0.635	0.545	−0.490	0.571
	10	0.815	0.700	−0.070	0.720
**Profile curvature**	−3–0	0.705	0.237	−1.370	0.299
	0.1–0.9	1.605	1.000	0.980	1.000
	1–2	0.425	0.000	−2.370	0.000
**SPI**	<50	3.205	1.000	1.880	1.000
	50.1–500	1.615	0.329	0.870	0.498
	500.1–2000	1.025	0.080	0.270	0.199
	2000.1–5000	0.835	0.000	−0.060	0.035
	>5000	0.975	0.059	−0.130	0.000
**Aspect**	Flat surfaces	3.645	1.000	1.560	1.000
	North	1.535	0.262	0.610	0.503
	North-East	1.095	0.108	0.130	0.251
	East	1.205	0.147	0.280	0.330
	South-East	1.605	0.287	0.690	0.545
	South	1.115	0.115	0.170	0.272
	South-West	0.785	0.000	−0.350	0.000
	West	1.015	0.080	0.040	0.204
	North-East	1.405	0.217	0.490	0.440
**Convergence index**	0.1–99	2.515	1.000	1.650	1.000
	−0.9–0	1.285	0.317	0.360	0.469
	−1.9–−1	0.915	0.111	−0.110	0.276
	−2.9–−2	0.715	0.000	−0.780	0.000
	−99–−3	0.985	0.150	−0.040	0.305
**HSG**	A	1.325	0.697	0.360	0.669
	B	1.595	1.000	0.890	1.000
	C	0.705	0.000	−0.710	0.000
	D	1.105	0.449	0.090	0.500

**Table 2:** Hardware and software environmental configuration used for modelling.

Configuration	Parameter
CPU	Intel(R) Core(TM) i7–7500@2.70 GHz
RAM	16.0 GB DDR4
GPU	NVIDIA GeForce MX330
Hard disk	SSD 512 GB M.2 PCIe
Operating system	Windows 10 Pro

**Table 3:** Statistical metrics used to evaluate model’s performance.

	Models	TP	TN	FP	FN	Sensitivity	Specificity	Accuracy	k-Index
**Training**	DLNN-FR	330	332	7	5	0.985	0.979	0.982	0.964
	DLNN-WOE	334	330	3	7	0.979	0.991	0.985	0.970
	ADT-FR	312	310	25	27	0.920	0.925	0.923	0.846
	ADT-WOE	309	311	28	26	0.922	0.917	0.920	0.840
**Validating**	DLNN-FR	132	128	12	16	0.892	0.914	0.903	0.806
	DLNN-WOE	137	128	7	16	0.895	0.948	0.920	0.840
	ADT-FR	129	124	15	20	0.866	0.892	0.878	0.757
	ADT-WOE	132	126	12	18	0.880	0.913	0.896	0.792

**Table 4:** The optimal parameters of the ADT based ensembles.

Models	No. of Iterations	Seed	Training Accuracy	Validating Accuracy
**ADT-FR**	23	6	0.923	0.878
**ADT-WOE**	28	8	0.920	0.896

## Data Availability

Data available on request.

## References

[1] Halkos G., Skouloudis A. (2020). Investigating resilience barriers of small and medium-sized enterprises to flash floods: A quantile regression of determining factors. Clim. Dev..

[2] Bezak N., Mikoš M. (2019). Investigation of Trends, Temporal Changes in Intensity-Duration-Frequency (IDF) Curves and Extreme Rainfall Events Clustering at Regional Scale Using 5 min Rainfall Data. Water.

[3] Bui D.T., Tsangaratos P., Ngo P.-T.T., Pham T.D., Pham B.T. (2019). Flash flood susceptibility modeling using an optimized fuzzy rule based feature selection technique and tree based ensemble methods. Sci. Total Environ..

[4] Cao C., Xu P., Wang Y., Chen J., Zheng L., Niu C. (2016). Flash flood hazard susceptibility mapping using frequency ratio and statistical index methods in coalmine subsidence areas. Sustainability.

[5] Costache R. (2019). Flash-Flood Potential assessment in the upper and middle sector of Prahova river catchment (Romania). A comparative approach between four hybrid models. Sci. Total Environ..

[6] Elkhrachy I. (2015). Flash flood hazard mapping using satellite images and GIS tools: A case study of Najran City, Kingdom of Saudi Arabia (KSA). Egypt. J. Remote Sens. Space Sci..

[7] Costache R., Bui D.T. (2020). Identification of areas prone to flash-flood phenomena using multiple-criteria decision-making, bivariate statistics, machine learning and their ensembles. Sci. Total Environ..

[8] Janizadeh S., Avand M., Jaafari A., Phong T.V., Bayat M., Ahmadisharaf E., Prakash I., Pham B.T., Lee S. (2019). Prediction Success of Machine Learning Methods for Flash Flood Susceptibility Mapping in the Tafresh Watershed, Iran. Sustainability.

[9] Hosseini F.S., Choubin B., Mosavi A., Nabipour N., Shamshirband S., Darabi H., Haghighi A.T. (2020). Flash-flood hazard assessment using ensembles and Bayesian-based machine learning models: Application of the simulated annealing feature selection method. Sci. Total Environ..

[10] Zhu Q. (2019). Research on Road Traffic Situation Awareness System Based on Image Big Data. IEEE Intell. Syst..

[11] Fu X., Pace P., Aloi G., Yang L., Fortino G. (2020). Topology Optimization Against Cascading Failures on Wireless Sensor Networks Using a Memetic Algorithm. Comput. Netw..

[12] Zhu J., Shi Q., Wu P., Sheng Z., Wang X. (2018). Complexity analysis of prefabrication contractors’ dynamic price competition in mega projects with different competition strategies. Complexity.

[13] Xiong L., Zhang H., Li Y., Liu Z. (2016). Improved stability and H∞ performance for neutral systems with uncertain Markovian jump. Nonlinear Anal. Hybrid Systems.

[14] Shi K., Wang J., Zhong S., Tang Y., Cheng J. (2020). Non-fragile memory filtering of TS fuzzy delayed neural networks based on switched fuzzy sampled-data control. Fuzzy Sets Syst..

[15] Xu M., Li T., Wang Z., Deng X., Yang R., Guan Z. (2018). Reducing complexity of HEVC: A deep learning approach. IEEE Trans. Image Process..

[16] Lv Z., Xiu W. (2019). Interaction of edge-cloud computing based on SDN and NFV for next generation IoT. IEEE Internet Things J..

[17] Chen H., Chen A., Xu L., Xie H., Qiao H., Lin Q., Cai K. (2020). A deep learning CNN architecture applied in smart near-infrared analysis of water pollution for agricultural irrigation resources. Agric. Water Manag..

[18] Chen H., Qiao H., Xu L., Feng Q., Cai K. (2019). A Fuzzy Optimization Strategy for the Implementation of RBF LSSVR Model in Vis–NIR Analysis of Pomelo Maturity. IEEE Trans. Ind. Inform..

[19] Qian J., Feng S., Li Y., Tao T., Han J., Chen Q., Zuo C. (2020). Single-shot absolute 3D shape measurement with deep-learning-based color fringe projection profilometry. Opt. Lett..

[20] Zhang S., Pak R.Y., Zhang J. (2020). Vertical time-harmonic coupling vibration of an impermeable, rigid, circular plate resting on a finite, poroelastic soil layer. Acta Geotech..

[21] Costache R., Zaharia L. (2017). Flash-flood potential assessment and mapping by integrating the weights-of-evidence and frequency ratio statistical methods in GIS environment–case study: Bâsca Chiojdului River catchment (Romania). J. Earth Syst. Sci..

[22] Khosravi K., Nohani E., Maroufinia E., Pourghasemi H.R. (2016). A GIS-based flood susceptibility assessment and its mapping in Iran: A comparison between frequency ratio and weights-of-evidence bivariate statistical models with multi-criteria decision-making technique. Nat. Hazards.

[23] Costache R., Hong H., Pham Q.B. (2020). Comparative assessment of the flash-flood potential within small mountain catchments using bivariate statistics and their novel hybrid integration with machine learning models. Sci. Total Environ..

[24] Tien Bui D., Khosravi K., Shahabi H., Daggupati P., Adamowski J.F., Melesse A.M., Thai Pham B., Pourghasemi H.R., Mahmoudi M., Bahrami S. (2019). Flood spatial modeling in northern Iran using remote sensing and gis: A comparison between evidential belief functions and its ensemble with a multivariate logistic regression model. Remote Sens..

[25] Razandi Y., Pourghasemi H.R., Neisani N.S., Rahmati O. (2015). Application of analytical hierarchy process, frequency ratio, and certainty factor models for groundwater potential mapping using GIS. Earth Sci. Inform..

[26] Siahkamari S., Haghizadeh A., Zeinivand H., Tahmasebipour N., Rahmati O. (2018). Spatial prediction of flood-susceptible areas using frequency ratio and maximum entropy models. Geocarto Int..

[27] Yang W., Xu K., Lian J., Ma C., Bin L. (2018). Integrated flood vulnerability assessment approach based on TOPSIS and Shannon entropy methods. Ecol. Indic..

[28] Razavi Termeh S.V., Pourghasemi H.R., Alidadganfard F. (2018). Flood Inundation Susceptibility Mapping using Analytical Hierarchy Process (AHP) and TOPSIS Decision Making Methods and Weight of Evidence Statistical Model (Case Study: Jahrom Township, Fars Province). J. Watershed Manag. Res..

[29] Dano U.L., Balogun A.-L., Matori A.-N., Wan Yusouf K., Abubakar I.R., Said Mohamed M.A., Aina Y.A., Pradhan B. (2019). Flood susceptibility mapping using GIS-based analytic network process: A case study of Perlis, Malaysia. Water.

[30] Khosravi K., Shahabi H., Pham B.T., Adamowski J., Shirzadi A., Pradhan B., Dou J., Ly H.-B., Gróf G., Ho H.L. (2019). A comparative assessment of flood susceptibility modeling using Multi-Criteria Decision-Making Analysis and Machine Learning Methods. J. Hydrol..

[31] Ali S.A., Parvin F., Pham Q.B., Vojtek M., Vojteková J., Costache R., Linh N.T.T., Nguyen H.Q., Ahmad A., Ghorbani M.A. (2020). GIS-based comparative assessment of flood susceptibility mapping using hybrid multi-criteria decision-making approach, naïve Bayes tree, bivariate statistics and logistic regression: A case of Topľa basin, Slovakia. Ecol. Indic..

[32] Chen W., Li Y., Xue W., Shahabi H., Li S., Hong H., Wang X., Bian H., Zhang S., Pradhan B. (2020). Modeling flood susceptibility using data-driven approaches of naïve bayes tree, alternating decision tree, and random forest methods. Sci. Total Environ..

[33] Chapi K., Singh V.P., Shirzadi A., Shahabi H., Bui D.T., Pham B.T., Khosravi K. (2017). A novel hybrid artificial intelligence approach for flood susceptibility assessment. Environ. Model. Softw..

[34] Avand M., Janizadeh S., Naghibi S.A., Pourghasemi H.R., Khosrobeigi Bozchaloei S., Blaschke T. (2019). A Comparative Assessment of Random Forest and k-Nearest Neighbor Classifiers for Gully Erosion Susceptibility Mapping. Water.

[35] Pham B.T., Prakash I., Bui D.T. (2018). Spatial prediction of landslides using a hybrid machine learning approach based on random subspace and classification and regression trees. Geomorphology.

[36] Choubin B., Moradi E., Golshan M., Adamowski J., Sajedi-Hosseini F., Mosavi A. (2019). An Ensemble prediction of flood susceptibility using multivariate discriminant analysis, classification and regression trees, and support vector machines. Sci. Total Environ..

[37] Wang Y., Hong H., Chen W., Li S., Panahi M., Khosravi K., Shirzadi A., Shahabi H., Panahi S., Costache R. (2019). Flood susceptibility mapping in Dingnan County (China) using adaptive neuro-fuzzy inference system with biogeography based optimization and imperialistic competitive algorithm. J. Environ. Manag..

[38] Costache R., Pham Q.B., Sharifi E., Linh N.T.T., Abba S., Vojtek M., Vojteková J., Nhi P.T.T., Khoi D.N. (2020). Flash-Flood Susceptibility Assessment Using Multi-Criteria Decision Making and Machine Learning Supported by Remote Sensing and GIS Techniques. Remote Sens..

[39] Bui D.T., Hoang N.-D., Martínez-Álvarez F., Ngo P.-T.T., Hoa P.V., Pham T.D., Samui P., Costache R. (2020). A novel deep learning neural network approach for predicting flash flood susceptibility: A case study at a high frequency tropical storm area. Sci. Total Environ..

[40] Arabameri A., Saha S., Chen W., Roy J., Pradhan B., Bui D.T. (2020). Flash flood susceptibility modelling using functional tree and hybrid ensemble techniques. J. Hydrol..

[41] Tehrany M.S., Pradhan B., Jebur M.N. (2014). Flood susceptibility mapping using a novel ensemble weights-of-evidence and support vector machine models in GIS. J. Hydrol..

[42] Prăvălie R., Costache R. (2014). The analysis of the susceptibility of the flash-floods’ genesis in the area of the hydrographical basin of Bāsca Chiojdului river/Analiza susceptibilitatii genezei viiturilor īn aria bazinului hidrografic al rāului Bāsca Chiojdului. Forum Geogr..

[43] Zarea R., Gheorghe M. (2010). Dangerous hydrological phenomena on the Hydrographic Basin Bâsca Chiojdului. Buletinul Institutului Politehnic Din Iaşi.

[44] Prăvălie R., Costache R. (2014). Assessment of socioeconomic vulnerability to floods in the Bâsca Chiojdului catchment area. Rom. Rev. Reg. Stud..

[45] Chen W., Li W., Chai H., Hou E., Li X., Ding X. (2016). GIS-based landslide susceptibility mapping using analytical hierarchy process (AHP) and certainty factor (CF) models for the Baozhong region of Baoji City, China. Environ. Earth Sci..

[46] Costache R. (2019). Flash-flood Potential Index mapping using weights of evidence, decision Trees models and their novel hybrid integration. Stoch. Environ. Res. Risk Assess..

[47] Skentos A. (2018). Topographic Position Index based landform analysis of Messaria (Ikaria Island, Greece). Acta Geobalcanica.

[48] Bui D.T., Pradhan B., Revhaug I., Tran C.T. (2014). A comparative assessment between the application of fuzzy unordered rules induction algorithm and J48 decision tree models in spatial prediction of shallow landslides at Lang Son City, Vietnam. Remote Sensing Applications in Environmental Research.

[49] De Rosa P., Fredduzzi A., Cencetti C. (2019). Stream Power Determination in GIS: An Index to Evaluate the Most’Sensitive’Points of a River. Water.

[50] Corrao M.V., Link T.E., Heinse R., Eitel J.U. (2017). Modeling of terracette-hillslope soil moisture as a function of aspect, slope and vegetation in a semi-arid environment. Earth Surf. Process. Landf..

[51] Zhang K., Ruben G.B., Li X., Li Z., Yu Z., Xia J., Dong Z. (2020). A comprehensive assessment framework for quantifying climatic and anthropogenic contributions to streamflow changes: A case study in a typical semi-arid North China basin. Environ. Model. Softw..

[52] Zhang K., Wang Q., Chao L., Ye J., Li Z., Yu Z., Yang T., Ju Q. (2019). Ground Observation-based Analysis of Soil Moisture Spatiotemporal Variability Across A Humid to Semi-Humid Transitional Zone in China. J. Hydrol..

[53] Singh C., Walia E., Kaur K.P. (2018). Enhancing color image retrieval performance with feature fusion and non-linear support vector machine classifier. Optik.

[54] Lin S.-W., Lee Z.-J., Chen S.-C., Tseng T.-Y. (2008). Parameter determination of support vector machine and feature selection using simulated annealing approach. Appl. Soft Comput..

[55] Lee S., Kim Y.-S., Oh H.-J. (2012). Application of a weights-of-evidence method and GIS to regional groundwater productivity potential mapping. J. Environ. Manag..

[56] Van Westen C. (1997). Statistical Landslide Hazards Analysis, ILWIS 2.1 for Windows Application Guide.

[57] Lee S., Pradhan B. (2007). Landslide hazard mapping at Selangor, Malaysia using frequency ratio and logistic regression models. Landslides.

[58] Nielsen M.A. (2015). Neural Networks and Deep Learning.

[59] Schmidhuber J. (2015). Deep learning in neural networks: An overview. Neural Netw..

[60] Agarap A.F. (2018). Deep learning using rectified linear units (relu). arXiv.

[61] Bui Q.-T., Nguyen Q.-H., Nguyen X.L., Pham V.D., Nguyen H.D., Pham V.-M. (2020). Verification of novel integrations of swarm intelligence algorithms into deep learning neural network for flood susceptibility mapping. J. Hydrol..

[62] Huang Z., Li J., Weng C., Lee C.-H. Beyond Cross-Entropy: Towards Better Frame-Level Objective Functions for Deep Neural Network Training in Automatic Speech Recognition. Proceedings of the INTERSPEECH—15th Annual Conference of the International Speech Communication Association.

[63] Goodfellow I., Bengio Y., Courville A. (2016). Deep Learning.

[64] Wu Y., Ke Y., Chen Z., Liang S., Zhao H., Hong H. (2020). Application of alternating decision tree with AdaBoost and bagging ensembles for landslide susceptibility mapping. Catena.

[65] Hong H., Pradhan B., Xu C., Bui D.T. (2015). Spatial prediction of landslide hazard at the Yihuang area (China) using two-class kernel logistic regression, alternating decision tree and support vector machines. Catena.

[66] Freund Y., Mason L. (1999). The Alternating Decision Tree Learning Algorithm. ICML.

[67] Khosravi K., Pham B.T., Chapi K., Shirzadi A., Shahabi H., Revhaug I., Prakash I., Bui D.T. (2018). A comparative assessment of decision trees algorithms for flash flood susceptibility modeling at Haraz watershed, northern Iran. Sci. Total Environ..

[68] Sahana M., Pham B.T., Shukla M., Costache R., Thu D.X., Chakrabortty R., Satyam N., Nguyen H.D., Phong T.V., Le H.V. (2020). Rainfall Induced Landslide Susceptibility Mapping Using Novel Hybrid Soft Computing Methods Based on Multi-layer Perceptron Neural Network Classifier. Geocarto Int..

[69] Wang S., Zhang K., van Beek L.P., Tian X., Bogaard T.A. (2020). Physically-based landslide prediction over a large region: Scaling low-resolution hydrological model results for high-resolution slope stability assessment. Environ. Model. Softw..

