# Correction: Costache et al. Flash-Flood Potential Mapping Using Deep Learning, Alternating Decision Trees and Data Provided by Remote Sensing Sensors. *Sensors* 2021, *21*, 280

**DOI:** 10.3390/s26061815

**Published:** 2026-03-13

**Authors:** Romulus Costache, Alireza Arabameri, Thomas Blaschke, Quoc Bao Pham, Binh Thai Pham, Manish Pandey, Aman Arora, Nguyen Thi Thuy Linh, Iulia Costache

**Affiliations:** 1 Research Institute of the University of Bucharest, 90-92 Sos. Panduri, 5th District, 050663 Bucharest, Romania; romulus.costache@icub.unibuc.ro; 2 National Institute of Hydrology and Water Management, București-Ploiești Road, 97E, 1st District, 013686 Bucharest, Romania; 3 Department of Geomorphology, Tarbiat Modares University, Tehran 36581-17994, Iran; 4 Department of Geoinformatics–Z_GIS, University of Salzburg, 5020 Salzburg, Austria; thomas.blaschke@sbg.ac.at; 5 Environmental Quality, Atmospheric Science and Climate Change Research Group, Ton Duc Thang University, Ho Chi Minh City 700000, Vietnam; 6 Faculty of Environment and Labour Safety, Ton Duc Thang University, Ho Chi Minh City 700000, Vietnam; 7 Geotechnical Engineering Deparment, University of Transport Technology, Hanoi 100000, Vietnam; binhpt@utt.edu.vn; 8 University Center for Research & Development (UCRD), Chandigarh University, Punjab140413, India; manish07sep@gmail.com; 9 Department of Civil Engineering, University Institute of Engineering, Chandigarh University, Punjab 140413, India; 10 Department of Geography, Faculty of Natural Sciences, Jamia Millia Islamia, New Delhi 110025, India; aman.jmi01@gmail.com; 11 Institute of Research and Development, Duy Tan University, Danang 550000, Vietnam; nguyentthuylinh58@duytan.edu.vn; 12 Faculty of Environmental and Chemical Engineering, Duy Tan University, Danang 550000, Vietnam; 13 Faculty of Geography, University of Bucharest, Bd. Nicolae Bălcescu No 1, 1st District, 010041 Bucharest, Romania; iulia.elena.costache@gmail.com

Following publication, concerns were raised regarding the relevance of a few references in this publication [1]. Adhering to our standard procedure, the Editorial Board conducted an investigation which determined that the original References 10–12,14,16–18,21–23,26,30,32,34,35 are irrelevant to the content of the article. As a result, following discussion with the authors, the Editorial Board has decided to remove References 10–12,14,16–18,21–23,26,30,32,34,35 from this publication [1]. With this correction, the order of some references has been adjusted accordingly.

**Text Correction**

1. In Section 2, Paragraph 2. The sentence “More information regarding the main flash floods occurred across the study area, as well as the damages caused by these phenomena, can be found in the research works carried out by: Costache and Zaharia [10], Prăvălie and Costache [57], Costache et al. [38], Zarea and Gheorghe [58], Prăvălie and Costache [59].” has been updated to “More information regarding the main flash floods that occurred across the study area, as well as the damages caused by these phenomena, can be found in the research works carried out by: Prăvălie and Costache [42], Costache et al. [23], Zarea and Gheorghe [43], Prăvălie and Costache [44].”

2. In Section 3.2, Paragraph 1. The sentence “For the study area, the map of slope angle was designed by splitting its range of values into five classes as following [12]: <3°; 3°–7°; 7.1°–15°; 15.1°–25°; >25° (Figure 2a).” has been updated to “For the study area, the map of slope angle was designed by splitting its range of values into five classes as follows: <3°; 3°–7°; 7.1°–15°; 15.1°–25°; >25° (Figure 2a).”

There is a typographic error in the Affiliation 3. The “Iranmailto” has been updated to “Iran”.

The authors state that the scientific conclusions are unaffected. This correction was approved by the Academic Editor. The original publication has also been updated.
